# Influence of the initial recurrence site on prognosis after radical surgery for colorectal cancer: a retrospective cohort study

**DOI:** 10.1186/s12957-023-03015-8

**Published:** 2023-04-27

**Authors:** Hongjiang Pu, Yaxue Chen, Ruoxia Shen, Yin Zhang, Duan Yang, Lizhu Liu, Xingxiang Dong, Guangjun Yang

**Affiliations:** 1 Department of Radiology, the Third Affiliated Hospital of Kunming Medical University, Yunnan Cancer Hospital, Yunnan Cancer Centre, Kunming, 650118 China; 2 Department of Oncology, Dazhou Central Hospital, Dazhou, 635000 Sichuan China; 3 Department of Nursing, Dazhou Vocational and Technical College, Dazhou, 635000 Sichuan China; 4 Department of Ultrasound Medicine, The Third Affiliated Hospital of Kunming Medical University, Yunnan Cancer Hospital, Yunnan Cancer Centre, Kunming, 650118 China

**Keywords:** Site of initial recurrence, Post-recurrence survival, Colorectal cancer, Prognosis

## Abstract

**Background & Aims:**

In this retrospective study, we aimed to elucidate how the initial recurrence site influences the post-recurrence survival (PRS) after the curative resection of colorectal cancer.

**Patients and methods:**

We collected samples from patients with stage I-III colorectal adenocarcinoma who were admitted to Yunnan Cancer Hospital from January 2008 to December 2019. Four hundred and six patients who developed recurrence after radical resection were included. The cases were classified according to the original site of recurrence as follows: liver metastases (*n* = 98), lung metastases (*n* = 127), peritoneum (*n* = 32), other individual organ (*n* = 69), two or more organs or sites (*n* = 49), and local recurrence (*n* = 31). Kaplan–Meier survival curves were used to compare the PRS of patients with different initial sites of recurrence. The influence of the initial recurrence site on PRS was analyzed using the Cox proportional hazards model.

**Results:**

The 3-year PRS of simple liver metastasis was 54.04% (95% CI, 45.46%-64.24%), and the 3-year PRS of simple lung metastasis was 50.05% (95% CI, 42.50%-58.95%). No significant difference was observed between simple liver metastasis or simple lung metastasis and local recurrence with a 3-year PRS of 66.99% (95% CI, 53.23%-84.32%). The 3-year PRS for peritoneal metastases was 25.43% (95% CI, 14.76%-43.82%), and the 3-year PRS for two or more organ sites was 34.84% (95% CI, 24.16%-50.24%). The peritoneal (hazard ratio [HR], 1.75; 95% CI, 1.10–2.79; *P* = 0.0189) and metastasis to two or more organs or sites (HR, 1.59; 95% CI, 1.05–2.43; *P* = 0.0304) were PRS-independent adverse prognostic factors.

**Conclusion:**

The prognosis of patients with peritoneum and multiple organs or sites recurred was poor. This study suggests early monitoring of peritoneal and multiple organ or site recurrence after surgery. This part of patients should receive comprehensive treatment as early as possible to improve their prognosis.

## Introduction

The key reason for the increasing mortality of colorectal cancer each year is the recurrence and metastasis of the cancer after radical surgery in some patients [1]. Data from our center published previously indicates that 25.10% of patients with stage I-III colorectal cancer experience postoperative recurrence [2]. Multiple influencing factors, including histological grade, serum CEA level, tumor location, surgical method, number of positive lymph nodes, and adjuvant chemotherapy, function as independent risk factors for the recurrence and metastasis of colorectal cancer post-surgery [3, 4]. A post-recurrence survival (PRS) of 23.1 months after colorectal cancer recurrence was reported, and the post-recurrence prognosis was found to be poor [5]. Older age, advanced pathological stage, rectal tumor, and recurrence are independent risk factors for PRS in colorectal cancer [6]. Postoperative recurrence surgery, chemotherapy, and radiotherapy can affect the PRS. The accurate detection of recurrent lesions after the resection of colorectal cancer and appropriate treatment post-recurrence can reduce the mortality of patients with colorectal cancer [7, 8].

Although the duration of survival post recurrence is significantly reduced, in recent years, with the advancement of medical technology, the methods used for oligometastatic therapy have been improved continuously, and currently, patients with post-surgery recurrence have longer survival. However, differences in the site of initial recurrence may still affect patient outcomes. The accurate detection of the site of initial recurrence leading to poor patient outcomes post recurrence is important for treatment. In addition, predicting the prognosis post recurrence is also important for determining the treatment strategy for colorectal cancer. However, a limited number of studies have investigated the effect of the initial site of recurrence on the postoperative prognosis.

In this study, the first recurrence of organ metastases after surgery was categorized based on six initial sites of recurrence, and the impact of each initial recurrence site on the post-recurrence prognosis was analyzed.

## Patients and methods

### Ethics statement

This study protocol was reviewed and approved by the Institutional Review Board of Yunnan Cancer Hospital (KY2019141) and conducted in accordance with the principles of the Declaration of Helsinki. Written informed consent was obtained from all patients.

### Inclusion of patients

The study was conducted in accordance with the requirements of the Reporting Epidemiology for Enhanced Observational Studies (STROBE) Statement: Guidelines for Reporting Observational Studies [9]. We included patients with stage I-III colorectal adenocarcinoma undergoing radical surgical resection who were treated at Yunnan Cancer Hospital from March 8, 2010 to May 29, 2018. Four hundred and six patients with postoperative recurrence and a follow-up of more than 3 years were included in this retrospective study. Patients who received preoperative neoadjuvant therapy and had a history of other primary tumors were excluded from this study.

### Postoperative monitoring and recurrence

Routine blood tumor marker testing and computed tomography (CT) scans of the thoracic and abdominal regions were performed every 3–6 months after 1–3 years and every 6–12 months after 4–5 years after colorectal cancer resection. When symptoms developed during the follow-up, imaging diagnosis was conducted based on the results of the following tests: positron emission tomography (PET-CT) or bone scintigraphy for bone pain; magnetic resonance imaging (MRI) or CT of the head for neurological symptoms; whole abdomen enhanced CT, abdominal ultrasound, and gastrointestinal endoscopy for abdominal symptoms. If the site of recurrence was identified, cranial MRI or CT and PET-CT or bone scintigraphy was routinely performed. Based on the results of the diagnostic imaging tests, collective diagnosis by a colorectal surgeon, medical oncologist, pathologist, and radiologist was performed to determine the site of recurrence. Needle biopsy was performed on the sites in which recurrence could not be confirmed.

### Classification of the site of initial recurrence

The site of initial recurrence was defined as the organ of recurrence and could be identified by each diagnostic imaging test performed before treatment for identifying recurrence. The initial metastatic organs were categorized based on six sites of initial recurrence: (1) simple liver metastasis; (2) simple lung metastasis, including metastasis in one or both lungs; (3) peritoneal metastasis (with or without abdominal organ metastasis); (4) metastasis to other individual organs, including an unlimited number of distant lymph nodes, bone, adrenal gland, ovary, uterus, bladder, ureter, abdominal wall, and muscles, among other organs; (5) two or more organs or sites; (6) local recurrence, clear diagnosis by colonoscopy biopsy, and no distant metastasis. Recurrence-free survival (RFS) and PRS were analyzed for patients with and without recurrence. RFS was defined as the survival from surgery to postoperative recurrence or all-cause death for colorectal cancer. PRS was defined as the time from first evidence detection to censored patient relapse to all-cause death in the absence of events in the last observation period.

### Statistical analysis

Continuous variables were compared between the two groups using the Mann–Whitney U test. Categorical variables were compared using Fisher's exact test. PRS was analyzed using the Kaplan–Meier method, and the log-rank test was used for intergroup comparisons. Univariate and multivariate analyses of PRS was performed using the Cox proportional hazards model. The following variables were analyzed: age, gender, body mass index, time to recurrence, initial recurrence site, surgical route, primary tumor location, tumor differentiation, tissue type, pathological T stage, pathological N stage, number of lymph nodes dissected, vessels, neural invasion, tumor deposition, postoperative adjuvant chemotherapy, adjuvant chemotherapy regimen, adjuvant chemotherapy cycle, palliative chemotherapy, preoperative carcinoembryonic antigen (CEA), carcinoma antigen 19–9 (CA199), and postoperative CEA. Multivariate analysis was performed for all variables with *P* value < 0.05 in the univariate analysis. The analyses were two-sided and conducted using the R software (version 3.6.3; http://www.R-project.org). Statistical significance was set at *P*-value < 0.05.

## Results

**Fig. 1:**
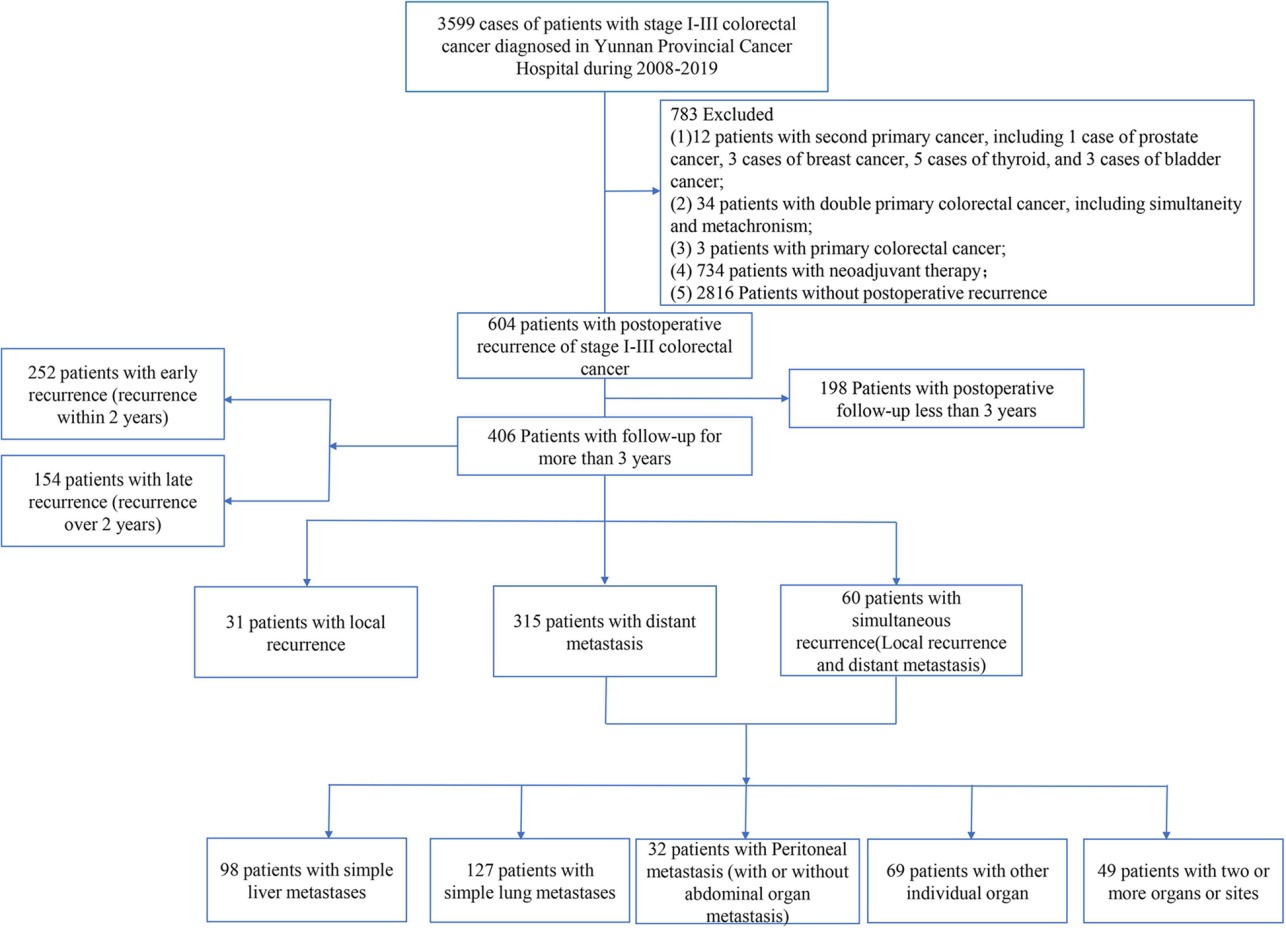
Study flow chart

A total of 3599 patients with stage I-III colorectal adenocarcinoma who underwent radical surgery in Yunnan Cancer Hospital from 2008 to 2019 were selected. In strict adherence with the inclusion and exclusion criteria, 406 patients with recurrence after radical resection were enrolled. According to recurrence within 2 years, 252 cases of early recurrence and 154 cases of late recurrence were identified. According to the recurrence pattern, 31 cases of local recurrence, 315 cases of distant metastasis, and 60 cases of simultaneous recurrence were identified. Based on the recurrence site, 98 cases (24.14%) of pure liver metastasis, 127 cases (31.28%) of pure lung metastasis, 32 cases (7.88%) of peritoneal metastasis, 69 cases (17.00%) of metastasis to other individual organs, and 49 cases (12.07%) of metastasis to two or more organ or sites were identified (Fig. 1). The median patient age was 60 years (range 21–87 years). Two hundred and twenty (54.19%) patients were males. The median RFS was 11.90 months (range 0.27–66.67 months). After at least 3 years of follow-up, 235 (57.88%) patients died (Table 1).

**Table 1:** Patient characteristics

Characteristic	All (406)	No death (171)	Death (235)	*P*-value
Age (years)				
Mean (SD)	58.72 (12.71)	58.11 (12.87)	59.17 (12.59)	0.408
Median (IQR)	60.00 (21.00–87.00)	60.00 (21.00–85.00)	61.00 (22.00–87.00)	
BMI (kg/m^2^)				
Mean (SD)	22.36 (3.25)	22.70 (3.24)	22.12 (3.23)	0.077
Median (IQR)	21.89 (13.89–34.29)	22.04 (15.24–31.60)	21.78 (13.89–34.29)	
Recurrence group				0.005
Local recurrence	31 (7.64%)	19 (11.11%)	12 (5.11%)	
Distant metastasis	315 (77.59%)	136 (79.53%)	179 (76.17%)	
Simultaneous recurrence	60 (14.78%)	16 (9.36%)	44 (18.72%)	
Recurrence time				0.058
Early recurrence	252 (62.07%)	97 (56.73%)	155 (65.96%)	
Late recurrence	154 (37.93%)	74 (43.27%)	80 (34.04%)	
Recurrence site				0.013
Liver	98 (24.14%)	47 (27.49%)	51 (21.70%)	
Lung	127 (31.28%)	52 (30.41%)	75 (31.91%)	
Peritoneum	32 (7.88%)	7 (4.09%)	25 (10.64%)	
Other individual organ	69 (17.00%)	31 (18.13%)	38 (16.17%)	
Two or more organs or sites	49 (12.07%)	15 (8.77%)	34 (14.47%)	
Local recurrence	31 (7.64%)	19 (11.11%)	12 (5.11%)	
Liver				0.179
No	308 (75.86%)	124 (72.51%)	184 (78.30%)	
Yes	98 (24.14%)	47 (27.49%)	51 (21.70%)	
Lung				0.747
No	279 (68.72%)	119 (69.59%)	160 (68.09%)	
Yes	127 (31.28%)	52 (30.41%)	75 (31.91%)	
Peritoneum				0.016
No	374 (92.12%)	164 (95.91%)	210 (89.36%)	
Yes	32 (7.88%)	7 (4.09%)	25 (10.64%)	
Other individual organ				0.604
No	337 (83.00%)	140 (81.87%)	197 (83.83%)	
Yes	69 (17.00%)	31 (18.13%)	38 (16.17%)	
Two or more organs or sites				0.082
No	357 (87.93%)	156 (91.23%)	201 (85.53%)	
Yes	49 (12.07%)	15 (8.77%)	34 (14.47%)	
Local recurrence				0.024
No	375 (92.36%)	152 (88.89%)	223 (94.89%)	
Yes	31 (7.64%)	19 (11.11%)	12 (5.11%)	
Sex, no. (%) of patients				0.738
Male	220 (54.19%)	91 (53.22%)	129 (54.89%)	
Female	186 (45.81%)	80 (46.78%)	106 (45.11%)	
Surgical approach				0.056
OR	291 (71.67%)	114 (66.67%)	177 (75.32%)	
LR	115 (28.33%)	57 (33.33%)	58 (24.68%)	
Primary site				0.102
Right colon	105 (25.86%)	36 (21.05%)	69 (29.36%)	
Left colon	85 (20.94%)	42 (24.56%)	43 (18.30%)	
Rectum	216 (53.20%)	93 (54.39%)	123 (52.34%)	
Tumor differentiation, no. (%) of patients				0.340
Unknown	39 (9.61%)	17 (9.94%)	22 (9.36%)	
Well	15 (3.69%)	6 (3.51%)	9 (3.83%)	
Moderate	199 (49.01%)	92 (53.80%)	107 (45.53%)	
Poor-undifferentiated	153 (37.68%)	56 (32.75%)	97 (41.28%)	
Mucinous type				0.260
No	386 (95.07%)	165 (96.49%)	221 (94.04%)	
Yes	20 (4.93%)	6 (3.51%)	14 (5.96%)	
T stage, no. (%) of patients				0.068
T0	12 (2.96%)	8 (4.68%)	4 (1.70%)	
T1	34 (8.37%)	18 (10.53%)	16 (6.81%)	
T3	336 (82.76%)	132 (77.19%)	204 (86.81%)	
T4	24 (5.91%)	13 (7.60%)	11 (4.68%)	
N stage, no. (%) of patients				< 0.001
N0	158 (38.92%)	87 (50.88%)	71 (30.21%)	
N1	149 (36.70%)	57 (33.33%)	92 (39.15%)	
N2	99 (24.38%)	27 (15.79%)	72 (30.64%)	
AJCC 8th ed. stage				< 0.001
I	39 (9.61%)	24 (14.04%)	15 (6.38%)	
II	119 (29.31%)	63 (36.84%)	56 (23.83%)	
III	248 (61.08%)	84 (49.12%)	164 (69.79%)	
Lymph node yield, n (%)				0.267
< 12	98 (24.14%)	46 (26.90%)	52 (22.13%)	
≥ 12	308 (75.86%)	125 (73.10%)	183 (77.87%)	
LVI				0.022
Unknown	54 (13.30%)	28 (16.37%)	26 (11.06%)	
Yes	56 (13.79%)	15 (8.77%)	41 (17.45%)	
No	296 (72.91%)	128 (74.85%)	168 (71.49%)	
PNI				0.270
Unknown	81 (19.95%)	34 (19.88%)	47 (20.00%)	
Yes	23 (5.67%)	6 (3.51%)	17 (7.23%)	
No	302 (74.38%)	131 (76.61%)	171 (72.77%)	
Tumor deposit, no. (%) of patients				0.191
No	340 (83.74%)	148 (86.55%)	192 (81.70%)	
Yes	66 (16.26%)	23 (13.45%)	43 (18.30%)	
Adjuvant chemotherapy, no. (%) of patients				0.635
No	109 (26.85%)	48 (28.07%)	61 (25.96%)	
Yes	297 (73.15%)	123 (71.93%)	174 (74.04%)	
Chemotherapy regimen				0.524
5-FU/capecitabine	25 (8.42%)	13 (10.57%)	12 (6.90%)	
CAPOX/XELOX	90 (30.30%)	34 (27.64%)	56 (32.18%)	
FOLFOX	164 (55.22%)	70 (56.91%)	94 (54.02%)	
Other	18 (6.06%)	6 (4.88%)	12 (6.90%)	
Chemotherapy cycle				0.748
< 6	153 (51.52%)	62 (50.41%)	91 (52.30%)	
≥ 6	144 (48.48%)	61 (49.59%)	83 (47.70%)	
Adjuvant radiotherapy				0.197
No	385 (94.83%)	165 (96.49%)	220 (93.62%)	
Yes	21 (5.17%)	6 (3.51%)	15 (6.38%)	
Palliative chemotherapy				0.581
No	32 (7.88%)	12 (7.02%)	20 (8.51%)	
Yes	374 (92.12%)	159 (92.98%)	215 (91.49%)	
Preoperative CEA, ng/mL				
Mean (SD)	31.22 (254.29)	13.14 (23.55)	44.34 (333.10)	0.225
Median (IQR)	5.33 (0.20–4688.00)	4.13 (0.49–150.50)	5.87 (0.20–4688.00)	
Preoperative CA19-9, ng/ml Mean				
(SD)	37.82 (90.08)	25.42 (36.39)	46.80 (113.46)	0.020
Median (IQR)	15.53 (0.59–1405.00)	14.80 (0.60–253.10)	16.78 (0.59–1405.00)	
Postoperative CEA, ng/mL Mean				
(SD)	34.36 (233.80)	5.55 (16.92)	55.41 (305.78)	0.042
Median (IQR)	2.38 (0.20–2965.00)	2.00 (0.20–174.60)	2.72 (0.20–2965.00)	

1 Data are presented as median (IQR), mean (SD), or n (%)

2 *Abbreviations*: *PRS* post-recurrence survival, *RFS* recurrence-free survival, *BMI* Body Mass Index, *CEA* carcinoembryonic antigen, *CA 19–9* carcinoma antigen 19–9, *LR* laparoscopic resection, *LVI* lymphovascular invasion, *OR* open resection, *PNI* perineural invasion

3 *P* value, using Wilcoxon Mann–Whitney test, chi-square test, or exact Fisher test depending on whether the variable is continuous or categorical

**Fig. 2:**
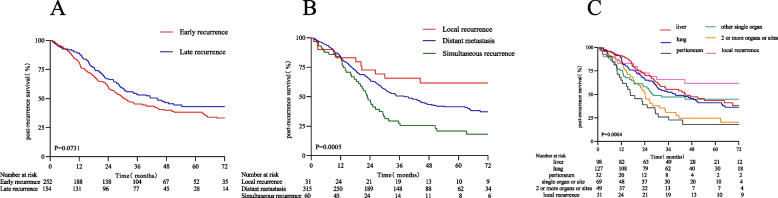
There was no significant difference in the PRS between patients with early recurrence and late recurrence (**A**). The PRS of synchronous recurrence was significantly lower than that of distant metastasis. Patients with local recurrence had the longest survival (**B**). The PRS of patients with metastasis to the peritoneum or two or more organs or sites was significantly lower than that of patients with recurrence in other sites (**C**). PRS: Post-recurrence survival rate

The 3-year PRS rate of 252 patients with early relapse was 44.59% (95% CI, 38.85%-51.19%), whereas the 3-year PRS rate of 154 patients with late recurrence was 53.28% (95% CI, 46.20%-61.45%) (HR, 0.78; 95% CI, 0.59–1.02; *P* = 0.0717) (Table 2 and Fig. 2A). The 3-year PRS of patients with local recurrence was 66.83% (95% CI, 53.04%-84.22%), and the 3-year PRS of patients with distant metastasis was 49.41% (95% CI, 44.16%-55.27%), which were significantly greater than the 3-year PRS in patients with synchronous recurrence and metastasis (30.32%; 95% CI, 21.10%-43.56%) (Table 2 and Fig. 2B). The 3-year PRS of patients with simple liver metastases was 54.04% (95% CI, 45.46%-64.24%), and the 3-year PRS of patients with simple pulmonary metastases was 50.05% (95% CI, 42.50%-58.95%). No significant difference was observed in patients with relapse. The 3-year PRS of patients with peritoneal metastases was 25.43% (95% CI, 14.76%-43.82%), and the 3-year PRS of patients with metastases to two or more organs was 34.84% (95% CI, 24.16%-50.24%) (Table 2 and Fig. 2C).

**Table 2:** Univariate and multivariate analyses of 3-year post-recurrence survival

Variables	3-year PRS (95% CI)	Univariate		Multivariate	
Recurrence group		HR (95% CI)	*P*-value	HR (95% CI)	*P*-value
Local recurrence	66.83 (53.04, 84.22)	1.0 (reference)		1.0 (reference)	
Distant metastasis	49.41 (44.16, 55.27)	1.75 (0.97, 3.15)	0.0618	1.19 (0.60, 2.38)	0.6185
Simultaneous recurrence	30.32 (21.10, 43.56)	2.96 (1.56, 5.62)	0.0009	2.17 (1.01, 4.64)	**0.0465**
Recurrence time					
Early recurrence	44.59 (38.85, 51.19)	1.0 (reference)			
Late recurrence	53.28 (46.20, 61.45)	0.78 (0.59, 1.02)	0.0717		
Recurrence site					
Liver	54.04 (45.46, 64.24)	1.0 (reference)			
Lung	50.05 (42.50, 58.95)	1.12 (0.79, 1.61)	0.5172		
Peritoneum	25.43 (14.76, 43.82)	2.23 (1.38, 3.60)	0.0011		
Other individual organ	46.26 (36.03, 59.40)	1.25 (0.82, 1.91)	0.2943		
Two or more organs or sites	34.84 (24.16, 50.24)	1.71 (1.10, 2.66)	0.0163		
Local recurrence	66.99 (53.23, 84.32)	0.65 (0.35, 1.22)	0.1829		
Liver					
No	46.00 (40.68, 52.01)	1.0 (reference)			
Yes	54.04 (45.46, 64.24)	0.80 (0.58, 1.09)	0.1526		
Lung					
No	47.03 (41.47, 53.34)	1.0 (reference)			
Yes	50.05 (42.50, 58.95)	0.92 (0.70, 1.22)	0.5704		
Peritoneum					
No	49.89 (44.96, 55.36)	1.0 (reference)		1.0 (reference)	
Yes	25.43 (14.76, 43.82)	1.97 (1.30, 2.98)	0.0015	1.75 (1.10, 2.79)	**0.0189**
Other individual organ					
No	48.29 (43.20, 53.99)	1.0 (reference)			
Yes	46.26 (36.03, 59.40)	1.06 (0.75, 1.50)	0.7323		
Two or more organs or sites					
No	49.68 (44.67, 55.26)	1.0 (reference)		1.0 (reference)	
Yes	34.84 (24.16, 50.24)	1.51 (1.04, 2.18)	0.0296	1.59 (1.05, 2.43)	**0.0304**
Local recurrence					
No	46.47 (41.59, 51.93)	1.0 (reference)		1.0 (reference)	
Yes	66.99 (53.23, 84.32)	0.53 (0.29, 0.94)	0.0310	0.48 (0.22, 1.03)	0.0582
Sex					
Male	46.60 (40.50, 53.62)	1.0 (reference)			
Female	49.51 (43.00, 57.00)	0.92 (0.71, 1.19)	0.5295		
Surgical approach					
OR	46.87 (41.46, 52.98)	1.0 (reference)			
LR	50.83 (42.56, 60.72)	0.89 (0.66, 1.20)	0.4556		
Primary site					
Right colon	41.69 (33.70, 51.57)	1.0 (reference)		1.0 (reference)	
Left colon	55.91 (46.70, 66.95)	0.66 (0.45, 0.98)	0.0370	0.67 (0.44, 1.01)	0.0538
Rectum	48.02 (41.85, 55.10)	0.84 (0.62, 1.13)	0.2419	0.87 (0.62, 1.24)	0.4475
Tumor differentiation					
Well	56.79 (38.27, 84.26)	1.0 (reference)			
Moderate	52.61 (46.24, 59.87)	1.13 (0.55, 2.33)	0.7300		
Poor-undifferentiated	41.62 (34.63, 50.03)	1.55 (0.75, 3.19)	0.2346		
Mucinous type					
No	48.48 (43.62, 53.89)	1.0 (reference)			
Yes	38.25 (23.05, 63.47)	1.33 (0.77, 2.28)	0.3054		
T stag					
T0	70.85 (50.49, 99.41)	1.0 (reference)			
T1	65.86 (53.18, 81.58)	1.21 (0.40, 3.66)	0.7313		
T3	44.65 (39.55, 50.41)	2.34 (0.87, 6.30)	0.0921		
T4	55.71 (39.35, 78.86)	1.70 (0.54, 5.34)	0.3646		
N stag					
N0	63.07 (56.29, 70.66)	1.0 (reference)		1.0 (reference)	
N1	44.06 (36.95, 52.53)	1.78 (1.30, 2.43)	0.0003	1.49 (1.04, 2.15)	**0.0315**
N2	26.55 (21.13, 38.57)	2.72 (1.95, 3.79)	< 0.0001	2.07 (1.41, 3.03)	**0.0002**
AJCC 8th ed. stage					
I	62.01 (52.16, 67.18)	1.0 (reference)			
II	48.63 (38.74, 54.83)	1.52 (0.85, 2.73)	0.1617		
III	23.57 (20.94, 34.72)	2.88 (1.66, 4.97)	0.0002		
Lymph node yield					
< 12	67.23 (59.97, 70.52)	1.0 (reference)			
≥ 12	59.82 (54.82, 68.51)	1.12 (0.82, 1.53)	0.4740		
LVI					
No	52.37 (49.98, 55.01)	1.0 (reference)		1.0 (reference)	
Yes	40.32 (38.92, 45.08)	2.01 (1.23, 3.29)	0.0054	1.46 (0.86, 2.50)	0.1628
PNI					
No	66.69 (60.83, 72.90)	1.0 (reference)			
Yes	62.75 (59.38, 68.47)	1.57 (0.90, 2.73)	0.1121		
TD					
No	59.21 (55.34, 62.07)	1.0 (reference)		1.0 (reference)	
Yes	49.10 (45.12, 52.55)	1.46 (1.05, 2.04)	0.0248	1.10 (0.74, 1.65)	0.6262
Adjuvant chemotherapy					
No	63.17 (57.82, 65.10)	1.0 (reference)			
Yes	55.86 (50.16, 60.04)	0.99 (0.74, 1.32)	0.9380		
Chemotherapy regimen					
5-FU/capecitabine	66.81 (61.90, 67.21)	1.0 (reference)			
CAPOX/XELOX	60.52 (58.42, 62.00)	1.62 (0.87, 3.03)	0.1284		
FOLFOX	53.26 (50.86, 65.33)	1.49 (0.82, 2.72)	0.1931		
Other	57.82 (51.78, 64.93)	1.80 (0.81, 4.01)	0.1510		
Chemotherapy cycle					
< 6	51.42 (48.26, 58.95)	1.0 (reference)			
≥ 6	59.71 (54.01, 63.29)	0.86 (0.64, 1.16)	0.3113		
Adjuvant radiotherapy					
No	59.28 (51.90, 62.77)	1.0 (reference)			
Yes	58.01 (51.00, 58.52)	0.68 (0.43, 1.07)	0.0950		
Palliative chemotherapy					
No	57.02 (49.72, 60.73)	1.0 (reference)			
Yes	55.88 (50.14, 58.92)	1.10 (0.84, 1.44)	0.5048		
Age group					
< 65	60.27 (53.93, 64.07)	1.0 (reference)			
≥ 65	57.82 (53.87, 62.05)	0.95 (0.72, 1.26)	0.7132		
BM group					
< 24	56.87 (48.97, 57.65)	1.0 (reference)		1.0 (reference)	
≥ 24	40.67 (31.98, 44.06)	1.46 (1.12, 1.89)	0.0046	1.12 (0.81, 1.54)	0.4850
Preoperative CEA group					
< 5	43.82 (39.92, 52.06)	1.0 (reference)		1.0 (reference)	
≥ 5	46.92 (41.77, 49.27)	1.44 (1.06, 1.95)	0.0183	1.29 (0.92, 1.80)	0.1336
Preoperative CA19-9 group					
< 37	49.82 (45.34, 54.37)	1.0 (reference)		1.0 (reference)	
≥ 37	30.62 (28.54, 42.45)	1.80 (1.35, 2.40)	< 0.0001	1.31 (0.93, 1.85)	0.1170

1 *Abbreviations*: *APR* abdominoperineal resection, *BMI* Body Mass Index, *CEA* carcinoembryonic antigen, *CA 19–9* carcinoma antigen 19–9, *LAR* low anterior resection, *LR* laparoscopic resection, *OR* open resection

Univariate and multivariate analyses of the factors associated with PRS are shown in Table 2. Univariate analysis showed that peritoneal metastasis, metastasis to two or more organs or sites, distant metastasis, right colon and rectal cancer, later pathological N stage, vascular invasion, tumor deposition, preoperative CEA, CA199 elevation, and postoperative CEA elevation were associated with a shorter PRS. Metastasis to the peritoneum (hazard ratio [HR], 1.75; 95% CI, 1.10–2.79; *P* = 0.0189) and two or more organs or sites (HR, 1.59; 95% CI, 1.05–2.43; *P* = 0.0189) in multivariate analysis (0.0304) along with a later pathological N stage was an independent poor prognostic factor for PRS (Table 2).

**Fig. 3:**
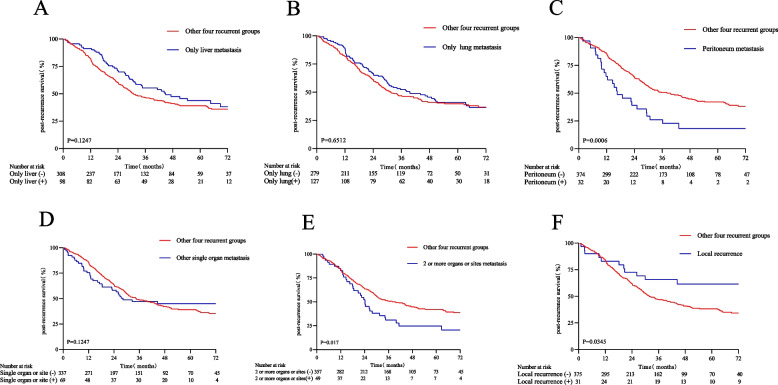
Patients with metastasis to the peritoneum or two or more organs or sites and local recurrence had worse PRS than patients without metastasis to the peritoneum or two or more organs or sites and local recurrence (**C**, **E**, and **F**). No significant differences were observed in the PRS of patients with or without recurrence at other sites (**A**, **B**, and **D**). PRS: Post-recurrence survival

The 3-year PRS rate was 25.43% (95% CI, 14.76%-43.82%) in patients with peritoneal recurrence, in contrast to 49.89% (95% CI, 44.96%-55.36%) in patients without peritoneal recurrence (HR, 1.97; 95% CI, 1.30–2.98; *P* = 0.0015) (Table 2 and Fig. 3C). The 3-year PRS rate was 34.84% (95% CI, 24.16%-50.24%) in patients with recurrence in two or more organ sites, whereas in patients with recurrence in other individual organs (including liver alone, lung alone, peritoneum, local metastasis, or metastasis in other sites), the 3-year PRS rate was 49.68% (95% CI, 44.67%-55.26%) (HR, 1.51; 95% CI, 1.04–2.18; *P* = 0.0296) (Table 2 and Fig. 3E). The 3-year PRS rate was 66.99% (95% CI, 53.23%-84.32%) in patients with local recurrence, compared with 46.47% (95% CI, 41.59%-51.93%) in patients without local recurrence (HR, 0.51; 95% CI, 0.29–0.94; *P* = 0.0310) (Table 2 and Fig. 3F). No significant difference was observed in the 3-year PRS between patients with and without liver metastases alone, lung metastases alone, and metastases to other individual organs (*P* > 0.05) (Fig. 3A, B, D).

## Discussion

The key finding of this study is that the prognosis of patients with local recurrence is the same as that of patients with liver or lung metastases alone. Patients with peritoneal metastases and metastases to two or more organ sites had the worst prognosis, and these were independent poor prognostic factors for PRS. Sawayama et al. demonstrated that liver metastases are associated with early recurrence in patients with stage I-III CRC [10]. Patients with hepatic recurrence with dissemination had a shorter PRS after recurrence compared with patients with recurrence in other organs. Patients with recurrence in the lungs had better prognosis than patients with recurrence in other organs. The number of metastatic organs and invasive treatment was correlated with patient prognosis. Future studies on the number of initial recurrences and treatment strategies for organs may help improve the prognosis of patients with colorectal cancer post recurrence.

In this study, we analyzed the data of patients who exhibited relapse within 2 years and those who exhibited relapse after 2 years and found no significant difference in the prognosis between the two groups. Possibly, 2 years is not the optimal cut-off value for assessing RFS. Evidence from studies has confirmed that colorectal cancer recurrence within 16 months of initial treatment should be marked as "early stage." A primary tumor stage of pT3,4/ypT3,4 and positive lymph node status pN + /ypN + predict early recurrence [11]. In other studies, patients with early recurrence of less than 13 months showed significantly shortened post-recurrence overall survival. Higher preoperative CA19-9 levels, venous invasion, and the absence of adjuvant chemotherapy were associated with early recurrence [12].

This study also showed that the incidence of pure lung metastases was higher than that of pure liver metastases. The potential reason is that we included 216 (53.20%) patients with rectal cancer. Patients with primary rectal cancer have been shown to be more likely to develop lung metastases than patients with colon cancer [13]. Clinical studies on colorectal cancer have shown differences in the metastatic patterns between mucinous adenocarcinoma, signet ring cell carcinoma, and the more common adenocarcinoma. Adenocarcinoma primarily metastasizes to the liver, whereas peritoneal metastasis is more common in mucinous adenocarcinoma and signet ring cell carcinoma [14]. Clinicopathological and prognostic differences between signet-ring cell carcinoma (SRCC) and adenocarcinoma (ADC) in colorectal cancer. SRCC is more likely than ADC to have a lymphatic and perineural invasion, resulting in significantly poorer survival outcomes. Improving the therapeutic effect of peritoneal metastasis may be the key to the treatment of SRCC[15].

When organs other than the liver were analyzed for metastases, the location was found to affect the metastatic patterns. Rectal cancers are more likely to metastasize to the chest, nervous system, and bones, whereas colon cancers are more likely to metastasize to the peritoneum [16]. The overall metastasis rate (including that of liver or lung metastasis) of right hemicolectomy has been shown to be lower. Meanwhile, left hemicolectomy has a higher rate of liver metastases. Sigmoidectomy has higher rates of liver, pulmonary, and brain metastases [17]. Patients with liver metastases from right-sided colon cancer have a worse prognosis than patients with liver metastases from left-sided colon cancer and are less likely to require re-excision [18]. The overall survival of patients with peritoneal recurrence was worse than that of patients with recurrence at other sites. In a study of 2077 patients with stage II or III colon cancer, female patients, stage T4, mucinous adenocarcinoma or signet ring cell carcinoma, and right colon cancer were associated with a significantly greater risk of postoperative peritoneal recurrence [19].

Some patients with post-recurrence metastatic colorectal cancer can be cured with surgery. Longer survival can be achieved by palliative chemotherapy, interventional therapy, radiofrequency treatment, and intraperitoneal hyperthermic perfusion chemotherapy. A careful follow-up and timely intervention of patients with colorectal cancer with progressive disease are essential components of the management strategy recommended by multidisciplinary treatment teams. Surgery should be combined with systemic chemotherapy in the treatment of colorectal cancer liver metastases [20]. Radiofrequency ablation of colorectal lung metastases can help achieve good long-term survival with a low incidence of serious adverse events [21]. Hyperthermic intraperitoneal chemotherapy (HIPEC) has emerged as a potential treatment modality for patients with intra-abdominal metastatic colorectal cancer, especially peritoneal metastases that can be cured by a combination of cytoreductive surgery and perioperative HIPEC [22]. Cytoreductive surgery and intraperitoneal hyperthermic chemotherapy (CRS-HIPEC) is increasingly being used to treat colorectal peritoneal metastasis (CPM). Significant learning curve (LC) improves perioperative outcomes after CRS-HIPEC for CPM [23]. Total mesorectal excision, or TME, demonstrates the pivotal role of regional lymphadenectomy in the surgical treatment of colorectal cancer. It has been reported that paraaortic lymph node dissection (PLND) has become a potentially effective treatment strategy for isolated paraaortic lymph node metastasis (PLNM) [24].

The only effective strategy for the long-term survival of patients with colorectal cancer with local recurrence is early detection of local recurrence and curative treatment [25]. Regardless of the advances in chemotherapy, the only cure for colorectal metastases is surgery, which necessitates complete resection from all metastatic sites [26].

This study has the following limitations. First, this study was a single-center retrospective study, which may have led to statistical bias in the results. Second, this study did not include cases of reoperation after recurrence. With the development of medical technology, patients with simple liver and lung metastases have recurrence and metastasis but can still achieve remission with surgical treatment. Third, this study did not include the palliative treatment plan and cycle of patients with unresectable metastatic rectal cancer, with or without standardized treatment and regular follow-up. Although various prognostic factors are present for patients with stage IV colorectal cancer, here, we included the maximum possible number of factors for a comprehensive analysis, and the results and conclusions of the analysis are credible.

In conclusion, the prognosis of patients with metastases to the peritoneum and multiple organs is poor. The findings of this study suggests the need for the early monitoring of peritoneal and multiple organ or site recurrence after surgery. Patients with recurrent peritoneal and multiple organ/site metastasis should receive comprehensive treatment as early as possible to improve their prognosis. Actively communicating with patients and their relatives or administering intensive treatment is necessary.

## Data Availability

Original data are available upon request to the corresponding author, Yang GJ.

## Abbreviations

PRS: Post-recurrence surviva
lRFS: Recurrence-free survival
CI: Confidence interval
CRC: Colorectal cancer
HR: Hazard ratio
IQR: Interquartile range
CEA: Carcinoembryonic antigen
CA 19-9: Carcinoma antigen 19–9

## References

[1] Fitzmaurice C, Abate D, Abbasi N, Abbastabar H, Abd-Allah F, Abdel-Rahman O (2019). Global, Regional, and National Cancer Incidence, Mortality, Years of Life Lost, Years Lived With Disability, and Disability-Adjusted Life-Years for 29 Cancer Groups, 1990 to 2017. JAMA Oncol.

[2] Li Z, Li S, Liang Y, Pu H, Tu C, Wu Z, You D (2020). Predictive Value of Postoperative Peripheral CD4+ T Cells Percentage in Stage I-III Colorectal Cancer: A Retrospective Multicenter Cohort Study of 1028 Subjects. Cancer Manag Res.

[3] Pu H, Xie P, Chen Y, Zhao Y, Ye X, Lu G, Zhang D, Li Z (2021). Relationship Between Preoperative and Postoperative Serum Carcinoembryonic Antigen and Prognosis of Patients with Stage I-III Rectal Cancer: A Retrospective Study of a Multicentre Cohort of 1022 Rectal Cancer Patients. Cancer Manag Res.

[4] Konishi T, Shimada Y, Hsu M, Tufts L, Jimenez-Rodriguez R, Cercek A, Yaeger R, Saltz L, Smith JJ, Nash GM, Guillem JG, Paty PB, Garcia-Aguilar J, Gonen M, Weiser MR (2018). Association of Preoperative and Postoperative Serum Carcinoembryonic Antigen and Colon Cancer Outcome. JAMA Oncol.

[5] Hassett MJ, Uno H, Cronin AM, Carroll NM, Hornbrook MC, Fishman P, Ritzwoller DP (2017). Survival after recurrence of stage I-III breast, colorectal, or lung cancer. Cancer Epidemiol.

[6] Safari M, Mahjub H, Esmaeili H, Abbasi M, Roshanaei G (2021). Specific causes of recurrence after surgery and mortality in patients with colorectal cancer: A competing risks survival analysis. J Res Med Sci.

[7] National-Comprehensive-Cancer-Network (NCCN) (2020). NCCN Clinical Practice Guidelines in Oncology (NCCN Guidelines): Colon Cancer. (Version 2.2020).

[8] National-Comprehensive-Cancer-Network (NCCN) (2020). NCCN Clinical Practice Guidelines in Oncology (NCCN Guidelines): Rectal Cancer. (Version 3.2020).

[9] von Elm E, Altman DG, Egger M, Pocock SJ, Gøtzsche PC, Vandenbroucke JP (2014). STROBE Initiative. The Strengthening the Reporting of Observational Studies in Epidemiology (STROBE) Statement: guidelines for reporting observational studies. Int J Surg.

[10] Sawayama H, Miyamoto Y, Hiyoshi Y, Ogawa K, Kato R, Akiyama T, Kiyozumi Y, Yoshida N, Baba H (2021). Overall survival after recurrence in stage I-III colorectal cancer patients in accordance with the recurrence organ site and pattern. Ann Gastroenterol Surg.

[11] Wiesmueller F, Schuetz R, Langheinrich M, Brunner M, Weber GF, Grützmann R, Merkel S, Krautz C (2021). Defining early recurrence in patients with resected primary colorectal carcinoma and its respective risk factors. Int J Colorectal Dis.

[12] Furuke H, Arita T, Kuriu Y, Shimizu H, Kiuchi J, Yamamoto Y, Konishi H, Morimura R, Shiozaki A, Ikoma H, Kubota T, Nakanishi M, Fujiwara H, Okamoto K, Otsuji E (2022). The survival after recurrence of colorectal cancer: a retrospective study focused on time to recurrence after curative resection. Surg Today.

[13] Robinson JR, Newcomb PA, Hardikar S, Cohen SA, Phipps AI (2017). Stage IV colorectal cancer primary site and patterns of distant metastasis. Cancer Epidemiol.

[14] Hugen N, van de Velde CJH, de Wilt JHW, Nagtegaal ID (2014). Metastatic pattern in colorectal cancer is strongly influenced by histological subtype. Ann Oncol.

[15] Wang L, Hirano Y, Heng G, Ishii T, Kondo H, Hara K, Obara N, Asari M, Kato T, Yamaguchi S (2021). Does signet ring cell carcinoma component signify worse outcomes for patients with colorectal cancer?. Asian J Surg.

[16] Riihimäki M, Hemminki A, Sundquist J, Hemminki K (2016). Patterns of metastasis in colon and rectal cancer. Sci Rep.

[17] Amri R, Bordeianou LG, Sylla P, Berger DL (2015). Variations in Metastasis Site by Primary Location in Colon Cancer. J Gastrointest Surg.

[18] Russolillo N, Sperti E, Langella S, Menonna F, Allieta A, Di Maio M, Ferrero A (2020). Impact of primary tumor location on patterns of recurrence and survival of patients undergoing resection of liver metastases from colon cancer. HPB (Oxford).

[19] Le VH, Thornblade L, Ituarte PHG, Lai LL, Melstrom KA (2021). Metachronous peritoneal metastases following curative resection for colon cancer: Understanding risk factors and patterns of recurrence. J Surg Oncol.

[20] Sugarbaker PH. Colorectal cancer: prevention and management of metastatic disease. Biomed Res Int. 2014;2014:782890. 10.1155/2014/782890. Epub 2014 Mar 24. 10.1155/2014/782890 PMC3982272 24783222

[21] Matsui Y, Hiraki T, Gobara H, Iguchi T, Fujiwara H, Nagasaka T, Toyooka S, Kanazawa S (2015). Long-term survival following percutaneous radiofrequency ablation of colorectal lung metastases. J Vasc Interv Radiol..

[22] Klempner SJ, Ryan DP (2021). HIPEC for colorectal peritoneal metastases. Lancet Oncol.

[23] Chidambarasamy ES, Chia CS, Johnny Ong CA, Soo KC, Ching Teo MC, Ching Tan GH (2022). Effect of the learning curve of cytoreductive surgery (CRS) and hyperthermic intraperitoneal chemotherapy (HIPEC) on the treatment of colorectal peritoneal metastasis. Asian J Surg.

[24] Sun KK, Wu YY (2021). Para-aortic lymph node dissection for colorectal cancer in the current era. Asian J Surg.

[25] Papachristodoulou A, Kouskos E, Markopoulos C, Karatzas G, Kouraklis G, Kostakis A (2002). Local recurrence after radical surgery for colorectal cancer. Int Surg..

[26] Abdalla EK, Adam R, Bilchik AJ, Jaeck D, Vauthey JN, Mahvi D (2006). Improving resectability of hepatic colorectal metastases: expert consensus statement. Ann Surg Oncol.

